# Nonlinear Optical Response of Au/CsPbI_3_ Quantum Dots and Its Laser Modulation Characteristics at 2.7 μm

**DOI:** 10.3390/mi15081043

**Published:** 2024-08-18

**Authors:** Mengqi Lv, Jin Zhao, Leilei Guo, Yanxu Zhang, Qiuling Zhao, Lihua Teng, Maorong Wang, Shuaiyi Zhang, Xia Wang

**Affiliations:** 1 Shandong Advanced Optoelectronic Materials and Technologies Engineering Laboratory, School of Mathematics and Physics, Qingdao University of Science & Technology, Qingdao 266061, China; 4022090007@mails.qust.edu.cn (M.L.); orangeade95@163.com (J.Z.); g98_0955@163.com (L.G.); 4022090010@mails.qust.edu.cn (Y.Z.); sdqlzhao@163.com (Q.Z.); tenglihua80@163.com (L.T.); shuaiyi163@163.com (S.Z.); 2 School of Physics and Technology, University of Jinan, Jinan 250022, China

**Keywords:** nonlinear optics, Er:YAP, perovskite, quantum dots

## Abstract

A passively Q-switched Er:YAP laser of 2.7 µm, utilizing Au-doped CsPbI_3_ quantum dots (QDs) as a saturable absorber (SA), was realized. It was operated stably with a minimum pulse width of 185 ns and a maximum repetition rate of 480 kHz. The maximum pulse energy and the maximum peak power were 0.6 μJ and 2.9 W, respectively, in the Q-switched operation. The results show that the CsPbI_3_ QDs SA exhibits remarkable laser modulation properties at ~3 μm.

## 1. Introduction

Based on the unique characteristics of the infrared spectral region, light sources that are related to mid-infrared band (2–5 μm) radiation are widely used in the laser imaging, medical treatment, and remote sensing fields [1,2]. There are important means of obtaining mid-infrared lasers, like optical parametric oscillators (OPOs), quantum cascade lasers (QCLs), solid-state lasers, and so on. Amidst them, solid-state lasers have garnered widespread attention because of their high power and excellent beam quality. At present, there are many active media doped with rare earth ions for generating laser radiation in the spectral range 2–3 μm, such as Er^3+^, Ho^3+^, etc., [3,4]. Generally, it can attain 2.1 μm laser emission by pumping Ho^3+^ ions [5] with a 1.9 μm laser or by co-pumping ions, which are co-doping Tm^3+^ and Ho^3+^ [6,7]. However, the overall conversion efficiency of this laser system is minimal. At present, 2.7–3 μm laser operation has been reported in various Er^3+^-doped laser crystals like Er: GSGG [8], Er: YSGG [9], Er: YLF [10], and Er: YAP [11], and so forth. Nevertheless, GSGG and YSGG crystals have low thermal conductivity and severe thermal effects, making it difficult to generate mid-infrared lasers with high repetition rates and high power. Although YLF possesses high thermal conductivity and stability, the crystal has high structure stress and thermal stress, and the crystal growth process is difficult. By contrast, Er:YAP exhibits lower phonon energy and higher thermal conductivity, which is beneficial for improving laser efficiency and reducing the thermal lensing effect. In addition, Er:YAP crystals can be grown by the Czochralski method [12].

Based on its excellent performance, Er:YAP pumped by LD has been widely applied to 2.7–3 μm laser outputs. In 2018, Quan et al. [13]. obtained dual-wavelength continuous- and pulsed-laser outputs of 2710 nm and 2728 nm by LD end-pumped Er:YAP crystals. In 2019, Yao et al. [14]. achieved a stable Q-switched pulse utilized for Er:YAP crystal lasers with dual wavelength outputs of 2730 nm and 2796 nm for the first time. In 2022, Cai et al. [15] realized a passively Q-switched mode-locked Er:YAP laser with an 800 ps pulse width at 3 μm using zirconium pentatelluride (ZrTe_5_) as a SA.

Perovskite quantum dots exhibit superior saturable absorption properties due to their unique physical and electronic structures, which show great potential for applications in generating passive mode-locked and Q-switched ultrafast laser pulses [16]. In 2016, Zhou et al. [17] inserted a layered CsPbBr_3_ nanocrystal film as a saturable absorber into a polarization-maintained ytterbium fiber laser to produce mode-locked pulses obtained with 216 ps and a maximum average output power of 10.5 mW. In 2021, Li et al. [18] successfully created a passive mode-locking laser by building a device which used a single piece of 2D CH_3_NH_3_PbI_3_ perovskite nanosheet. More and better research is coming out [19,20]. As for solid-state lasers, and it is more challenging to obtain high-quality Q-switched or mode-locked pulses.

In this paper, Au-doped CsPbI_3_ QDs were prepared and characterized without inert gas protection. A passively Q-switched 2.9 μm Er:YAP laser with a Au-doped CsPbI_3_ QDs SA was operated successfully at room temperature. The shortest pulse duration, of 185 ns, the maximum pulse energy, of 0.6 μJ, and the peak power, of 2.9 W, were obtained.

## 2. Material Synthesis and Characterization

### 2.1. Preparation and Characterization of CsPbI_3_ QDs

Unlike the traditional hot-injection method, the CsPbI_3_ QDs were fabricated without inert gas protection [21]. At the first step, a proper amount of PbI_2_ precursor solution was injected into a beaker, which was heated with a stirrer until the temperature reached 120 °C. Then, the preheated Cs-oleate (Cs-OL) precursor solution was quickly introduced into the beaker, and then we let it cool down in an ice-water bath. After that, the prepared CsPbI_3_ QDs were dispersed in n-hexane after centrifugation. In addition, one can obtain a CsPbI_3_-Au solution by mixing a prepared gold nanoparticle n-hexane solution with a CsPbI_3_ QDs solution.

**Figure 1:**
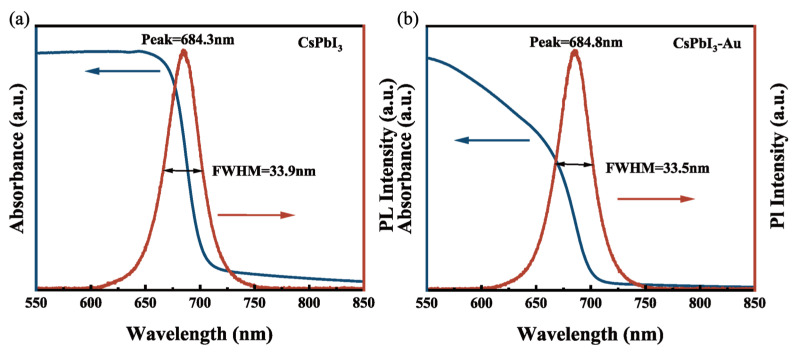
(**a**) Normalized photoluminescence spectrum (PL) (red line) and absorption spectrum (blue line) for the CsPbI_3_ perovskite QDs’ dispersion; (**b**) CsPbI_3_-Au perovskite QDs.

As shown by the red line in Figure 1a,b, photoluminescence (PL) spectra were recorded by the microscopic measurement system equipped with a spectrometer (USB4000, Ocean Optics, Delray Beach, FL, USA), and the wavelength of the pump laser source was 360 nm. The center wavelength of PL spectra was 684.3 nm with a full width at half maxima (FWHM) of 33.9 nm in the inset of Figure 1a. Au-doped CsPbI_3_ QDs showed a slight red shift (0.5 nm) due to the large size of the gold nanoparticles. The FWHM of Au-doped CsPbI_3_ QDs was 0.4 nm smaller than the CsPbI_3_ QDs, which may be due to the agglomeration effect of large particles, but the size distribution range was smaller. The UV–visible absorption spectra of the CsPbI_3_ and the CsPbI_3_-Au, respectively, as determined by the UV-visible-near-infrared (NIR) spectrophotometer (Cary5000), are represented by the blue lines in Figure 1a,b. A comparison of the two figures demonstrates the material’s absorption changes when gold nanoparticles were present.

**Figure 2:**
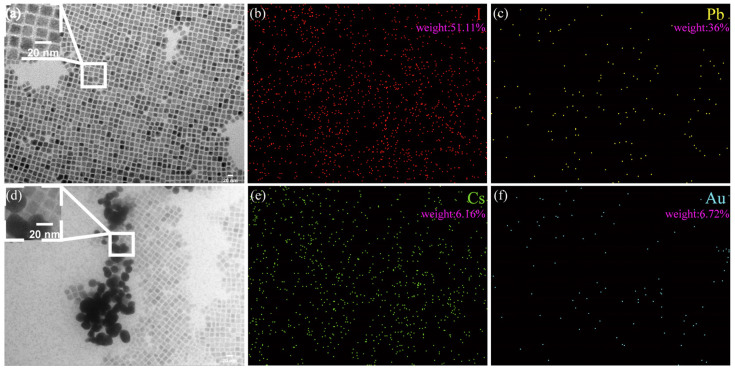
(**a**) Transmission electron microscopy (TEM) image of CsPbI_3_ perovskite QDs and (**d**) CsPbI_3_-Au perovskite QDs; elemental mapping of (**b**) I, (**c**) Pb, (**e**) Cs, and (**f**) Au.

Figure 2a,d separately show the transmission electron microscope (TEM, HT7800, Hitachi, Tokyo, Japan) image of the colloidal QDs of CsPbI_3_ and Au-doped CsPbI_3_. The energy dispersive spectroscopy (EDS) elemental maps reveal that the compositional distributions of four elements (I (b), Pb (c), Cs (e), and Au (f)) in the CsPbI_3_-Au perovskite QDs are uniform.

**Figure 3:**
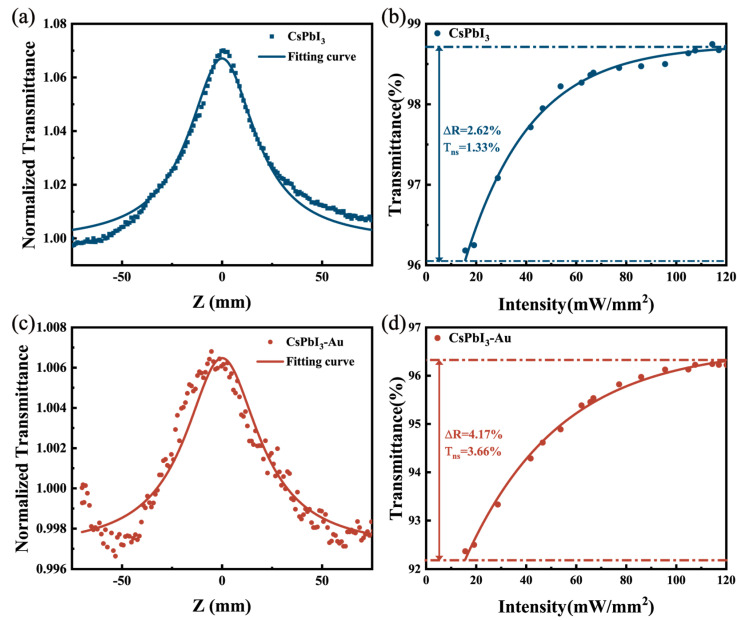
(**a**,**c**) Open-aperture Z-scan experimental results of CsPbI_3_ perovskite QDs and CsPbI_3_-Au perovskite QDs, respectively, (**b**,**d**) and nonlinear transmission versus intensity of CsPbI_3_ and CsPbI_3_-Au, respectively.

The saturation absorption properties of CsPbI_3_ and CsPbI_3_-Au were investigated using an open-aperture Z-scan system. The light source of the Z-scan experiment was a Femto-second laser with a 400 nm central wavelength frequency, doubling from 800 nm fundamental light (Spectra-Physics, Milpitas, CA, USA). The pulse duration of the laser was 60 fs, and the repetition frequency was 80 MHz. The data of Z-scan experimental are shown in Figure 3a,c, and the experimental data are fitted by the following formula [22].

(1) $$T\left(Z\right)=\sum_{n=0}^{\infty \;}\frac{{\left(-q_{0}\right)}^{n}}{{\left(n+1\right)}^{3/2}{\left(1+Z^{2}/{Z_{0}}^{2}\right)}^{n}}$$

where the freedom facto $q_{0}=\beta L_{eff}I_{0}$, $Z_{0}$ is the diffraction length of the beam, the effective length $L_{eff}=1-e^{-\alpha L}/\alpha$, $I_{0}$ is the maximum on-axis intensity at the focal point, α is the linear absorption coefficient, and L denotes the thickness of the sample. The nonlinear absorption coefficient β of CsPbI_3_ and CsPbI_3_-Au, −1.54 × 10^−6^ cm/W and −2.07 × 10^−6^ cm/W, can be calculated, respectively.

As shown in Figure 3b,d, the nonlinear transmittance of CsPbI_3_ and CsPbI_3_-Au increases with the incident optical intensity. The experimental data could be fitted by the following formula [23]:

(2) $$T=1-∆R\cdot exp\left(-\frac{I}{I_{s}}\right)-T_{ns}$$

Here, ∆R is the modulation depth, $I_{s}$ is the saturation intensity, and $T_{ns}$ is nonsaturable losses. The saturation intensity, modulation depth, and nonsaturable loss of CsPbI_3_ were 117.1 mW/mm^2^, 2.62%, and 1.33%. The saturation intensity, modulation depth, and nonsaturable loss of CsPbI_3_-Au were 135.3 mW/mm^2^, 4.17%, and 3.66%.

### 2.2. Properties of CsPbI_3_ QDs Saturable Absorber

The CsPbI_3_ SA was prepared by spin-coating. Firstly, the CsPbI_3_ colloidal QDs material was ultrasonically dispersed for 10 min. After that, 20 µL of the material was dripped on the YAG substrate and dried in a cool and ventilated place for 10 min to ensure that the CsPbI_3_ colloidal quantum dot material formed a thin film on the substrate, so as to obtain the CsPbI_3_ saturable absorption mirror (CsPbI_3_-SA). The CsPbI_3_-Au saturable absorption mirror (CsPbI_3_-Au-SA) was prepared by the same method. The transmittance characteristics of CsPbI_3_ SA and gold-doped CsPbI_3_ SA in the range of 800–3300 nm were measured by an ultraviolet–visible near-infrared spectrophotometer (Cary5000, Agilent, Santa Clara, CA, USA).

**Figure 4:**
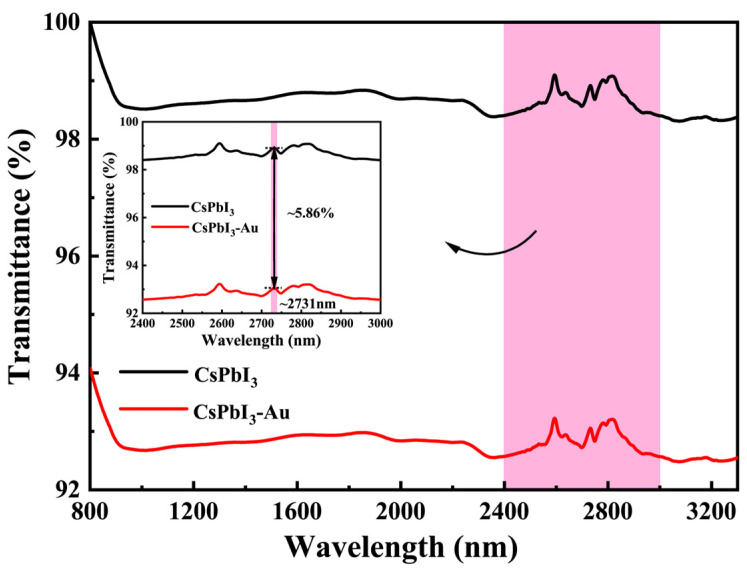
Transmittance of CsPbI_3_ QDs SA and Au-doped CsPbI_3_ SA at 2.7 μm; inset: magnified view of transmittance of CsPbI_3_ QDs SA and Au-doped CsPbI_3_ SA in the range of 2400–3000 nm.

As shown in Figure 4, the transmission of CsPbI_3_ and Au-doped CsPbI_3_ in the YAG substrate is 98.9% and 93.1%, respectively, around ~2.7 μm. The transmission of Au-doped CsPbI_3_ presents as 5.86% lower than that of the CsPbI_3_ SA, and the absorption of light in the near-infrared band is enhanced after gold doping [24]. In the near-infrared Q-switched experiment, compared with the CsPbI_3_ SA, gold-doped CsPbI_3_ SA can increase the loss of the resonant cavity and output laser pulses with a narrower pulse width and higher pulse energy.

## 3. Results and Discussion

**Figure 5:**
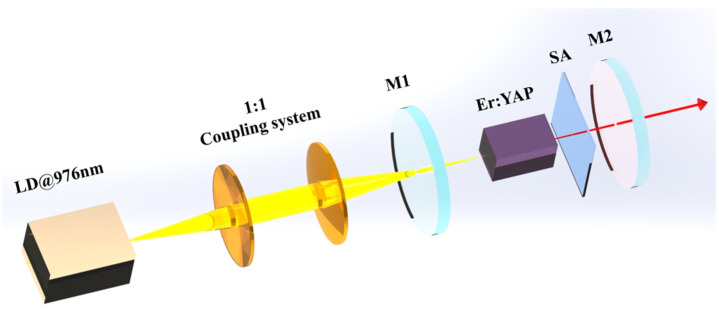
Experimental scheme of the passively Q-switched Er:YAP laser based on the CsPbI_3_ Au-doped QDs SA.

As shown in Figure 5, the experimental device of passively Q-switched Er:YAP laser was described in Refs. [25,26]. The pumping source (BWT, Beijing, China) employed a semiconductor laser with a fiber diameter of 105 μm and a center wavelength of 976 nm. With a 1:1 optical coupling system, the pump beam was focused into the crystal. The cavity length of the resonant cavity is about 25 mm. The laser crystal was YAP crystal doped with 10 at. % Er^3+^ with a size of 2 × 2 × 6 mm^3^. When the crystal was excited by a high-energy laser, the refractive index changed with temperature, leading to the thermal lens effect [27]. Therefore, the crystal was cooled by water circulation at 10 °C, and the copper radiator was applied to accelerate the heat dissipation of the crystal wrapped in indium foil. The experimental setup is shown in Figure 5; one side of the laser crystal is a flat mirror with high reflection (HR), coated at 2700 nm, and high transmittance (HT), coated at 976 nm, as an end mirror of the resonator. Two types of concave mirrors with two transmittances ($T_{OC}=$ 1% and 4%) and a radius of curvature of −100 mm are used as output mirrors. The laser output power and spectrum were recorded using a power meter (THORLABS, S302C, Newton, NJ, USA) and a spectrometer (THORLABS, OSA207C), respectively.

**Figure 6:**
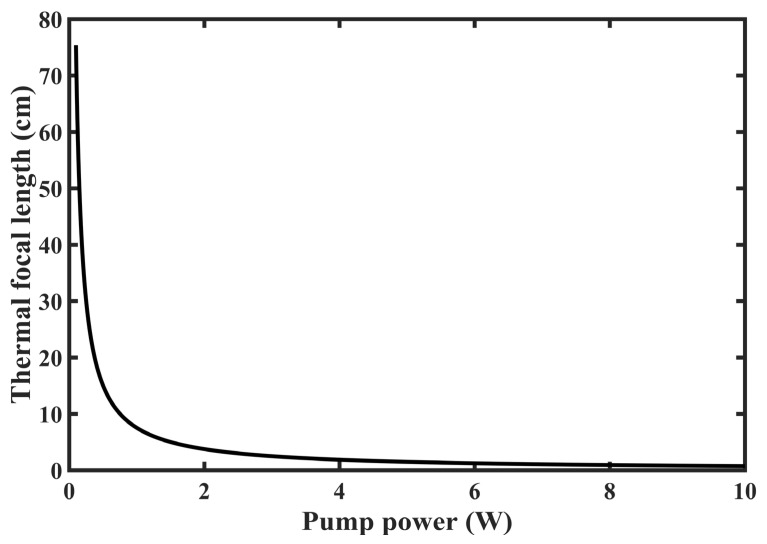
Thermal focal length of Er:YAP crystal versus pump power.

The thermal focal lengths of Er:YAP crystals at different absorbed pump powers were simulated using the Matlab R2024a program, as shown in Figure 6. The Er:YAP crystals are infinite when the absorbed pump power tends to 0, and the radius of curvature of the crystals decreases with the increase in the absorbed pump power.

**Figure 7:**
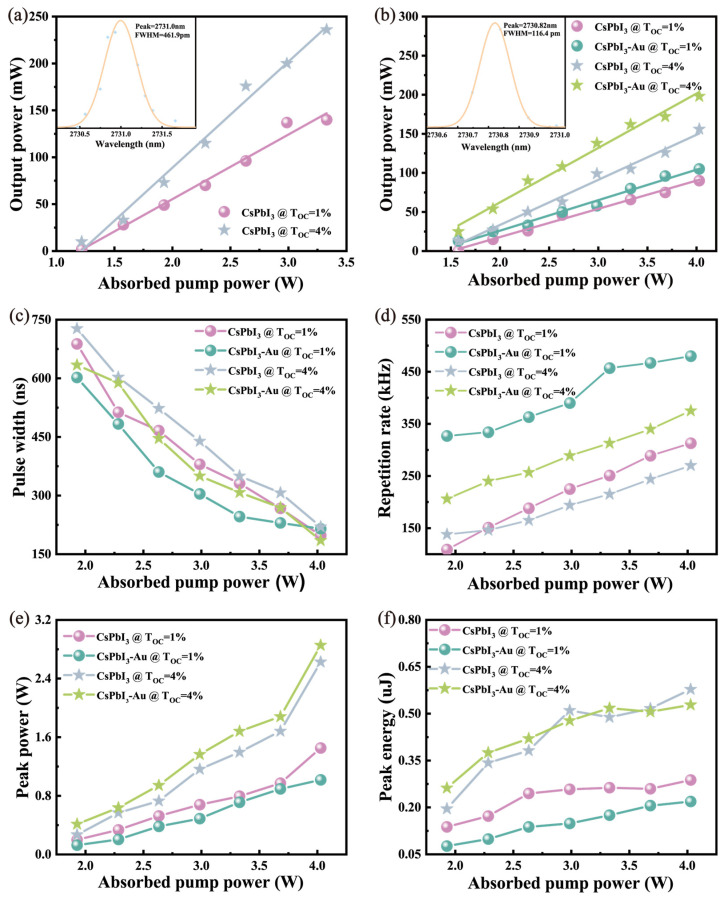
(**a**) Average output power of the Er:YAP laser versus various pump power for continuous wave (CW) operation using $T_{oc}$ = 1% and 4%; (**b**) average output power of the Q-switched operation versus diverse incident power using $T_{oc}$ = 1% and 4%; inset of (**a**,**b**) the laser spectrum at wavelengths of 2731.0 nm and 2730.8 nm, respectively; the passively Q-switched Er:YAP laser related parameters versus the absorbed pump power, (**c**) pulse width, (**d**) repetition rate, (**e**) peak power, and (**f**) pulse energy correspond to different saturated absorbers.

The pump power threshold of the laser was 1.8 W under the output coupling mirror with $T_{oc}$ = 1%, as shown in Figure 7a in the CW output mode. Correspondingly, when the laser power was 1.8 W, the output power equipped with $T_{oc}$ = 4% was higher than $T_{oc}$ = 1%. With the pump power increasing, the maximum average output power attained 236 mW with a slope efficiency of 11.31% and a light-to-light conversion efficiency of 7.09%. The pumping power is only increased to 4.6 W in order to protect the crystal. Further increases in pump power led to instability in the Q-switching operation.

The threshold pump power of the laser was 2.6 W in the resonant cavity with the CsPbI_3_ SA using $T_{oc}$ = 1%. Figure 7c,d show that the relevant parameters measured under 4.6 W pump power include pulse width, repetition rates, peak power, and pulse energy. The pulse width was 198 ns and the repetition frequency was 313 kHz, corresponding to an average output power of 90 mW, a slope efficiency of 3.61%, and a light-to-light conversion efficiency of 2.23%. Compared with the resonator without nanomaterials, the loss of the resonator was evidently increased [28]. The threshold pump power of laser oscillation was 2.2 W in the Au-doped CsPbI_3_ SA using $T_{oc}$ = 1%. Under the output power of 4.6 W, the pulse width and the repetition rates were, respectively, 215 ns and 480 kHz, corresponding to the average output power, slope efficiency, and light-to-light conversion efficiency, which were 105 mW, 3.90%, and 2.61%, respectively.

When CsPbI_3_ SA was inserted into the laser device with $T_{oc}$ = 4%, the threshold of pump power reached 2.2 W, which is lower than $T_{oc}$ = 1%. The pulse width and the repetition rates were 220 ns and 270 kHz, respectively, matching along with an average output power of 156 mW, a slope efficiency of 5.77%, a light-to-light conversion efficiency of 3.87%, and a $T_{oc}$ of 4%. The pump power threshold was lower when choosing an Au-doped CsPbI_3_ SA, and the pulse width was 185 ns, which reached the minimum value in this work. The values of repetition rate, average output power, slope efficiency, and light-to-light conversion efficiency were 375 kHz, 198 mW, 6.96%, and 4.92%, separately. The loss of the resonator using a $T_{oc}$ of 1% is less than a $T_{oc}$ of 4%, and the time required for the laser oscillation formation is shorter, so the pulse repetition rate is higher, which is consistent with our experimental data. The data explicitly show that the CsPbI_3_ QDs with gold have a higher output power, a narrower pulse width, and a stronger pulse energy relative to the CsPbI_3_ QDs without gold [29]. We compared the current research results on passive Q-switched of Er:YAP crystal infrared lasers, as shown in Table 1 below.

**Figure 8:**
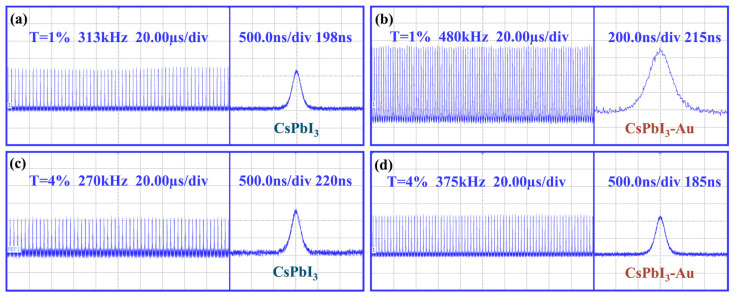
Passively Q-switched pulse trains and single waveform in pulse trains of (**a**) CsPbI_3_ SA and (**b**) Au-doped CsPbI_3_ SA using $T_{oc}$ = 1%; (**c**) CsPbI_3_ SA and (**d**) Au-doped CsPbI_3_ SAs using $T_{oc}$ = 4% under pump power of 4.6 W.

As can be seen from Figure 8, the output pulse was detected by the photodetector (VIGO, pps03) and the digital oscilloscope (Agilent Technologies, Santa Clara, CA, USA, DSO-X3104A) under 4.6 W pump power.

The scanning times of the digital oscilloscope were 20 μs/div and 500 ns/div, respectively. It is evident that the single pulse waveform and the pulse trains obtained from the Au-doped CsPbI_3_ SA were narrower and more stable compared with the passively Q-switched Er:YAP experiment in graphene SAs [34]. When the transmittance of the output coupling mirror was 4% and Au-doped nanomaterials were used, the narrowest pulse width of the output was 185 ns. Meanwhile, it had a maximum peak power of 2.9 W and a pulse energy of 0.5 μJ. In the experiments, the maximum peak power densities on the saturable absorbers CsPbI_3_ and CsPbI_3_-Au were 925 W/mm^2^ and 893 W/mm^2^, respectively. At this time, there was no damage to the CsPbI_3_ and CsPbI_3_-Au saturable absorbers, so the damage thresholds for CsPbI_3_ and CsPbI_3_-Au were greater than 925 W/mm^2^ and 893 W/mm^2^, respectively. This suggests that CsPbI_3_ and CsPbI_3_-Au have the potential to produce higher power ultrafast lasers.

**Figure 9:**
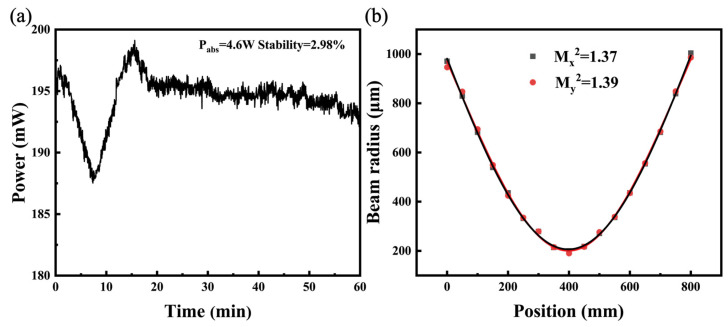
(**a**) Average output power fluctuations over time. (**b**) Beam quality of a passively Q-switched Er:YAP laser at an absorbed pump power of 4.6 W.

To assess the stability of the average output power of the Q-switched laser, we used a power meter to record the power over time, as shown in Figure 9a. The average output power values were systematically recorded at one-second intervals over the course of an hour. It is noteworthy that the observed variation in average output power remained around 2.98% when the absorbed pump power (P_abs_) was kept at 4.6 W. The average output power of the Q-switched laser was also found to be stable over time, as shown in Figure 9a. We ran the Q-switched laser for seven hours, and the pulse profile observed on the oscilloscope remained stable. When the laser was switched off and restarted after a week, subsequent oscilloscope checks confirmed the good stability of the Q-switched laser in our experiments. The laser beam quality at a pump power of 4.6 W was determined by measuring the beam radius after passing through the focusing lens (f = 400 mm). This is shown in Figure 9b. The beam radius of the laser was recorded using the 90/10 knife method. The $M_{x}^{2}$ and $M_{y}^{2}$ factors are calculated to be 1.37 and 1.39, respectively, and the correspongding far-field divergence angles are 23.8 mrad and 25.5 mrad.

## 4. Conclusions

Perovskite quantum dots of CsPbI_3_ doped with Au nanoparticles were synthesized at room temperature, which were prepared as the saturable absorbers based on the highly nonlinear absorption properties. Utilizing the Au-doped CsPbI_3_ SA, a passive Q-switched Er:YAP laser at 2730.82 nm was operated stably. The maximum average output power of 198 mW, the greatest peak power of 2.9 W, and the minimum pulse width of 185 ns were achieved, respectively. The results show that doped perovskite quantum dots exhibit the potential of infrared laser modulation.

## Figures and Tables

**Table 1:** Comparison of property of passively Q-switched Er:YAP solid-state laser realization.

Er:YAP	SA	Slope Efficiency [%]	Pulse Width [ns]	Repetition Rate [kHz]	Ref
5 at. %	Gold nanorods	6.4	313.2	196.8	[28]
5 at. %	Graphene	13	460	114	[30]
5 at. %	Zn:C_3_N_4_		162.5	192.9	[31]
10 at. %	PtSe_2_	14	141.8 ± 1.4	296.2 ± 3.8	[32]
10 at. %	ZrTe_5_	4.2	169	446	[15]
10 at. %	NiV-LDH	6.9	141	295	[25]
10 at. %	NiCo-LDH	5.8	230	198	[25]
10 at. %	SnSe_2_	7.18	198	317	[26]
10 at. %	ReSe_2_	14.8	202.8	244.6	[14]
15 at. %	TaSe_2_	11.5	264	105.5	[33]
10 at. %	CsPbI_3_	5.77	198	313	This work
10 at. %	CsPbI_3_-Au	6.96	185	480	This work

## Data Availability

The original contributions presented in the study are included in the article, further inquiries can be directed to the corresponding authors.
